# Intra-cervical Foley Balloon Catheter Versus Prostaglandins for the Induction of Labour: A Literature Review

**DOI:** 10.7759/cureus.33855

**Published:** 2023-01-17

**Authors:** Biswambhar Sangram Singh, Ketav Joshi, Sandhya Pajai

**Affiliations:** 1 Obstetrics and Gynecology, Jawaharlal Nehru Medical College, Datta Meghe Institute of Higher Education and Research, Wardha, IND

**Keywords:** foley's catheter, dinoprostone, cervical ripening, prostaglandins, labour induction

## Abstract

Labour induction involves helping a woman to start her labour, before labour begins on its own, for a vaginal birth with the aid of artificial methods, such as medications or other medical techniques. Labour induction is done in cases where extending the pregnancy can threaten the mother or her baby's health, and delivery should result in better outcomes than continuing the pregnancy. Currently, nearly 25% of babies are born by labour induction in economically developed countries. It is often necessary in certain situations to induce labour by using ripening techniques that not only soften the cervix but also make it thin and dilated. Mechanical or pharmacological approaches are used for the artificial induction of labour. Because research articles evaluating the safety and efficacy of various ripening techniques of the cervix vary in terms of their findings, it remains uncertain as to which is the best way to induce labour. In light of this, to find out the most popular interventions for ripening of the cervix during labour induction, we performed a review of the literature that compares the use of a Foley catheter and prostaglandins (misoprostol and dinoprostone). Our findings show that using misoprostol orally is much better than using it vaginally. Foley catheter proved to be the least effective induction technique, despite the fact that it offers the lowest risk.

## Introduction and background

The use of artificial procedures that induce labour before the commencement of spontaneous labour and after the age of fetal viability is known as labour induction. Ripening of the cervix is crucial for successful labour induction. A sequence of biochemical processes that result in lower levels of collagen and glycosaminoglycans and an increase in water content soften and dilate the cervix throughout normal pregnancy, making it more conducive to natural labour or labour induction [1]. If this process does not succeed in a normal pregnancy, artificial ripening of the cervix is necessary. Cervical ripening and artificial labour induction have been used for centuries [2]. Currently, in more economically developed countries, nearly 25% of all babies are born by labour induction [3]. This process is chosen if the benefits of induction outweigh the risk of continuing a pregnancy [4]. In the absence of a not-so-favourable cervix, administering Pitocin or rupturing of membranes artificially is unlikely to induce labour successfully [5]. In these situations, it is often necessary to induce labour using ripening techniques that not only soften the cervix but also make it thin and dilated. Mechanical or pharmacological approaches are used for labour induction. A popular technique used for labour induction is placing a mechanical tool like a Foley catheter inside the cervical canal. This method was first described for labour induction in 1967 and is more cost-efficient than other mechanical procedures that had been used previously [6].

Examples of pharmacological techniques for inducing labour include the usage of prostaglandin, oxytocin, oestrogens, and mifepristone. In obstetrics and gynaecology, prostaglandins, which are cyclopentane derivatives of arachidonic acid, are frequently employed [7]. The only prostaglandin that the Food and Drug Administration (FDA) of the United States has approved for ripening of the cervix during labour induction is prostaglandin E2 (PGE2), also known as dinoprostone. Dinoprostone needs to be stored in cool temperatures and is expensive [8]. Misoprostol is a prostaglandin E1 (PGE1) analogue that has long been recommended to treat gastric ulcers brought on by non-steroidal anti-inflammatory drugs (NSAIDs) and is frequently used off-label to induce labour. Although the efficacy of misoprostol in the ripening of the cervix has been long established, some case reports have revealed that the frequency of significant consequences, such as uterine hyper-contractility and rupture, may be higher when compared to other techniques [9]. Because the findings of research articles evaluating the safety and efficacy of various ripening techniques of the cervix vary, it remains uncertain as to which is the best way to induce labour. Hence, in order to find out the most popular techniques for ripening the cervix during labour induction, we conducted a literature review that compares using a Foley catheter, misoprostol, and dinoprostone. Additionally, with a view to contributing to improving clinical practice and assisting with the design of the subsequent trials, we engaged in a thorough assessment of the available research [10].

## Review

Methodology

Data Sources

**Figure 1:**
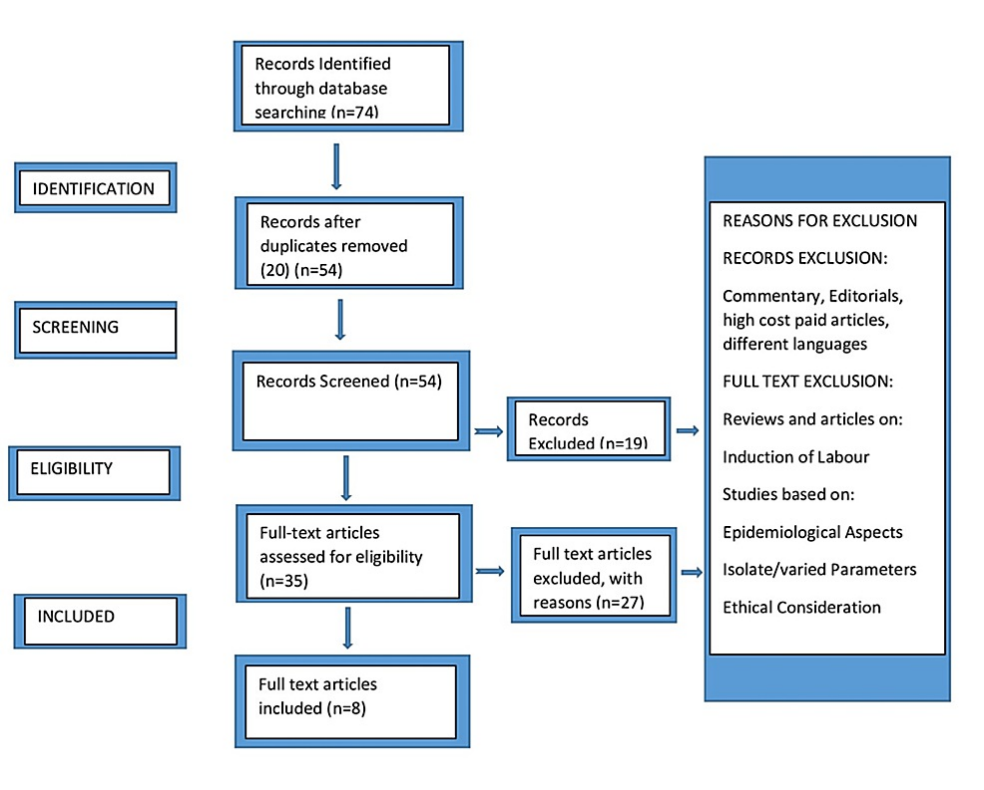
PRISMA flowchart depicting the study selection procedure PRISMA: Preferred Reporting Items for Systematic Reviews and Meta-Analyses

We looked for available research articles on cervical ripening or labour induction published up to 2022 with the help of keywords like "labour induction," "prostaglandin," and "foley catheter" on PubMed, Scopus, and Google Scholar databases. Around 74 articles were identified, out of which 20 articles were removed as they were duplicates. Finally, after a full-text article screening, eight articles were finalized to be reviewed. The reference list of related articles was likewise thought to be a reliable source of knowledge. No experiments were carried out to discover the unreported data (Figure 1). The exclusion criteria were as follows: a history of preterm birth, antepartum hemorrhage, placenta lying low, previous cesarean sections, current HSV infection, poly or oligohydramnios, hypertension, and any chronic illness or condition that would make the use of prostaglandin contraindicated. The procedures covered in this literature review are Foley catheter, intracervical dinoprostone, oral misoprostol, and vaginal misoprostol. We concentrated on three outcomes: induction-to-delivery interval, uterine overstimulation, and rate of cesarean delivery.

Background

Labour induction starts the process of cervical thinning, uterine dilatation, and contraction, which leads to a baby being delivered artificially. When extending the pregnancy poses a risk to the health of the mother or her unborn child, it aims to end the pregnancy by a conventional vaginal delivery. The results of the delivery should be preferable to those of continuing the pregnancy. When labour is medically induced if the cervix is not mature, it will lead to challenges for the mother and fetus. Local insertion via Foley catheter, or PGE2 is regularly utilized, and numerous research has been conducted to determine the effectiveness in reducing the rate of cesarean deliveries [11-13]. The first step in the induction of labour in females with a not-so-favorable cervix is cervical ripening, which can be accomplished mechanically with a Foley catheter and, if done pharmacologically, can be achieved with PGE1 or PGE2 analogs. Over the past three decades, prostaglandins have largely replaced mechanical methods as the primary means of inducing labour in high-resource environments [13]. When asked about the delivery expectations of pregnant women, one of the main things they wanted is for their labour to be brief or short-lasting [14]. According to a questionnaire-based study that assessed how women view their birthing experiences regarding labour induction, a long induction-to-delivery interval appeared to be a significant determinant in patients' dissatisfaction with the delivery process [15]. Prolonged labour is directly linked to higher chances of postpartum fever, infections in newborns, and maternal chorioamnionitis. Therefore, when selecting a strategy for inducing labour, the induction-to-delivery interval should be considered [16]. We studied a total of eight articles, and the participants predominantly comprised singleton pregnancies in the third trimester of pregnancy with a cervix that happened to be unfavorable, intact membranes, and cephalic presentation of the fetus. And our study mainly focused on three outcomes: time between induction and delivery, uterine hyperstimulation, and frequency of cesarean delivery.

Induction-to-delivery interval

According to the studies by Mizrachi et al., Edwards et al., and Barda et al., the time between induction and delivery was lower in the Foley catheter group when compared with dinoprostone or misoprostol groups [17,18,19,20]. However, the study published by Henry et al. and Jozwiak et al. stated that the time between induction and delivery was faster with the usage of PGE2 [21]. Nevertheless, when there was a comparison between Foley+PGE2 and Foley catheter alone, the induction-to-delivery interval was shorter with the Foley+PGE2 group, as reported by Chowdhary et al. This is a significant finding as neither strategy resulted in hyperstimulation or infection but significantly decreased the induction-to-delivery interval with a similar Cesarean section rate [22,23]. Also, the time between induction and delivery was lower with Foley+PGE2 insert when compared with intravaginal PGE2 alone. We also studied a meta-analysis published by Chen et al., and it was found the time interval between induction and delivery was lower with misoprostol when compared with a Foley catheter; they reported that misoprostol is the best drug for faster induction of delivery [24]. However, none of these research articles mentioned the Foley catheter size, which we consider to be a major drawback.

Cesarean delivery rate

The study by Chen et al. reported that the probability of cesarean section was lowest with the use of oral misoprostol. All the other studies failed to show any marked difference in Cesarean delivery rate. However, among all the randomized controlled trials (RCT) we studied, Jozwiak et al.'s had the largest sample size, and in that study, they compared the use of PGE2 gel with the use of Foley catheter and found that cesarean section rate was similar in both. However, two major adverse events were also noted in the PGE2 gel group: perforation of the uterus and rupture of the uterus [18,20,23].

Uterine hyperstimulation

In the studies this article reviewed, there was a non-significant increase in the rate of uterine hyper-stimulation with the use of PGE2 when compared to Foley catheters. The survey done by Jozwiak et al. reported that the usage of a Foley catheter during induction appeared to reduce hyperstimulation of the uterus and postpartum bleeding, but this was also statistically insignificant. Also, the study by Chen et al. reported that the best method for preventing uterine hyperstimulation was the use of a Foley catheter, and the likelihood of vaginal misoprostol is considered a bad treatment method concerning hyperstimulation of the uterus and fetal heart alterations [20,23]. Another interesting study by Garg et al. stated that the hyperstimulation of the uterus was greater in the Foley+misoprostol group as compared to the Foley+dinoprostone group. But this finding was also statistically insignificant [13]. All in all, it can be suggested that the PGE1 analog misoprostol is the worst drug as far as uterine hyperstimulation is considered. However, the usage of a Foley catheter in addition to misoprostol increased the risk of chorioamnionitis. This may be due to the more frequent vaginal inspections required to administer misoprostol in repeated dosages [23]. However, the study by Garg et al. reported no such cases of chorioamnionitis, and this might be attributed to a single, one-time placement of prostaglandins, which may be intracervical or intravaginal. There are shreds of evidence to suggest that repeated doses of prostaglandins plus Foley increased the risk of chorioamnionitis, and hence the one-time placement of prostaglandin is advised [14,25,26,27,28] (Table 1). 

**Table 1:** Studies comparing different induction techniques PGE2: prostaglandin E2

Study	Statistical groups	Observed parameters	Inference
Mizrachi et al., Israel, 2016 [18]	346 nulliparous females were equally divided into two study groups. The 1st group underwent induction of labour (IOL) using a Foley catheter, and the 2nd group underwent induction through PGE2	Cesarean delivery rate, induction-delivery interval, amniotic fluid with meconium stain, oxytocin augmentation use, adverse maternal outcomes, and unfavorable neonatal outcomes	Induction to delivery was faster with a Foley catheter. Higher requirement for oxytocin augmentation in the Foley catheter group. Peripartum cord pH was slightly lesser in the PGE2 group. No change in the cesarean delivery rate
Garg et al., Chandigarh, India, 2021 [14]	150 women were divided equally into two study groups. 1st group underwent IOL using misoprostol+Foley. The 2nd group underwent IOL using dinoprostone+Foley	Induction-delivery interval, change in the Bishop score, cesarean section rate, uterine hyperstimulation, and chorioamnionitis	No significant difference was observed in induction-to-delivery time and cesarean section rate. Change in the Bishop score was higher with the Foley+misoprostol group. No significant difference in uterine hyperstimulation. No cases of chorioamnionitis were observed
Chowdhary et al., Chandigarh, India, 2019 [23]	110 women were divided equally into two study groups. Group 1 underwent IOL using an intracervical Foley. Group 2 underwent using Foley+PGE2 gel	Induction-delivery interval, cesarean delivery rate, uterine overstimulation, and chorioamnionitis	Induction to delivery was faster with Foley+PGE2 gel. No difference in the cesarean section rate. No uterine hyperstimulation or chorioamnionitis case was seen
Eser et al., Germany, 2018 [32]	294 women were categorized into two groups. Group 1 underwent IOL using intravaginal PGE2 alone (n=148). Group 2 used Foley+PGE2 insert (n=146)	Induction-to-delivery time, duration of the latent phase	Induction to delivery was faster in group 2. The latent phase was more significant in group 1
Edwards et al., Birmingham, Alabama, US, 2014 [19]	376 women were randomized into two groups. Group 1 underwent IOL with a Foley catheter (n=185). Group2 underwent IOL with dinoprostone (n=191)	Induction-to-delivery time. The number of females delivering within 24 hours. Cesarean section rate	Induction-to-delivery time was shorter with the Foley catheter. More patients were delivered within 24 hours in the case of a Foley catheter. Cesarean delivery rate was more with dinoprostone, but the data was statistically non-significant
Jozwiak et al., Netherlands, 2011 [21]	819 women were randomized into two groups. Group 1 (n=411) underwent IOL with a Foley catheter, and group 2 (n=408) underwent IOL with prostaglandin E2	Cesarean delivery rate. The time between induction to delivery. Uterine hyperstimulation. Arterial cord pH. Oxytocin augmentation	Induction to delivery was faster with PGE2. IOL with PGE2 required lower oxytocin augmentation. No difference in the cesarean section rate
Barda et al., Israel, 2017 [20]	300 women were equally randomized into two groups: n=150 for each group. The first group underwent IOL with a Foley catheter and the second group underwent IOL with dinoprostone	The time interval between induction to delivery. Induction of labour within 24 hours. Oxytocin augmentation. Cesarean delivery rate. Neonatal complications	The Foley catheter group reached active labour in a significantly shorter time. The need for oxytocin augmentation was more significant in the Foley catheter group. The difference in cesarean section rate was statistically non-significant. Both groups showed no difference in neonatal outcomes
Henry et al., Australia, 2013 [22]	101 women were randomized into two groups. The first group (n=50) underwent IOL with a Foley catheter and the second group (n=51) underwent IOL with PGE2	Vaginal delivery within 12 hours of induction. Vaginal delivery rate. Cesarean section rate. Oxytocin augmentation. The interval between the ripening of the cervix and vaginal delivery	Vaginal delivery within 12 hours was more with the usage of PGE2. The Foley catheter group required more oxytocin augmentation. The interval between the ripening of the cervix and vaginal delivery was shorter with PGE2

Limitations

This review has some limitations that warrant discussion. Firstly, we found that the evaluated research was quite heterogeneous. The main clinical heterogeneities pertain to the experience of inserting the research agent, the volume of the Foley catheter used, and the maximum timeframes from inserting the labour-inducing agent to removing it. Also, because relatively fewer study parameters were included in this analysis, subgroup analysis for these heterogeneities was not conducted. Moreover, we only included articles published in English and failed to discuss many other clinical scenarios such as mortality of the mother and perinatal death as these outcomes were not reported in the clinical trials. Evidence suggests that larger Foley catheters (60 or 80 ml) are more effective than smaller ones (30 ml) and that 24-hour Foley catheters are less effective than 12-hour ones [29-31].

## Conclusions

In terms of the induction-to-delivery interval, the most efficient ways to induce labour in women with intact membranes after 28 weeks of pregnancy are vaginal misoprostol and dinoprostone; however, these methods are associated with an increased risk of uterine hyperstimulation and other complications like uterine perforation. The least successful induction technique is the use of a Foley catheter, but it also had the lowest risk of uterine hyperstimulation and alterations in the fetal heart rate. Oral misoprostol is a more efficient approach than vaginal misoprostol in terms of reducing the chance of a cesarean section and generating less uterine hyperstimulation with fetal heart rate variations. Additionally, it is advised that only one prostaglandin insert is used while using misoprostol in combination with Foley because repeated doses of prostaglandins in combination with Foley have been demonstrated to increase the risk of chorioamnionitis and uterine hyperstimulation. However, as described earlier, this study has a few limitations that warrant serious discussion.
